# Optical fibre sensors: their role in in vivo dosimetry for prostate cancer radiotherapy

**DOI:** 10.1186/s12645-016-0020-y

**Published:** 2016-10-18

**Authors:** P. Woulfe, F. J. Sullivan, S. O’Keeffe

**Affiliations:** 1 Optical Fibre Sensors Research Centre, University of Limerick, Limerick, Ireland; 2 Department of Radiotherapy Physics, Galway Clinic, Galway, Ireland; 3 Prostate Cancer Institute, National University of Ireland Galway, Galway, Ireland; 4 Department of Radiotherapy, Galway Clinic, Galway, Ireland

**Keywords:** Radiotherapy, Brachytherapy, Optical fibre, Dosimetry, Radiation sensor, Radioluminescence

## Abstract

Review is made of dosimetric studies of current optical fibre technology in radiotherapy for therapeutic applications, focusing particularly on in vivo dosimetry for prostate radiotherapy. We present the various sensor designs along with the main advantages and disadvantages associated with this technology. Optical fibres are ideally placed for applications in radiotherapy dosimetry; due to their small size they are lightweight and immune to electromagnetic interferences. The small dimensions of optical fibres allows it to be easily guided within existing brachytherapy equipment; for example, within the seed implantation needle for direct tumour dose analysis, in the urinary catheter to monitor urethral dose, or within the biopsy needle holder of the transrectal ultrasound probe to monitor rectal wall dose. The article presents the range of optical fibre dosimeter designs along with the main dosimetric properties required for a modern in vivo dosimetry system to be utilised in a clinical environment.

## Background

Dosimetry in radiotherapy has developed dramatically in recent years with evolving technology in a period of extensive clinical development. New radiation technologies have been developed and adopted for clinical use in prostate cancer treatment in response to a need to deliver dose-escalated radiation therapy while minimising treatment-related morbidity. This has occurred with the introduction of new advanced treatment techniques, for example, volumetric modulated arc therapy (VMAT), flattening filter free (FFF) beams and the introduction of Magnetic Resonance Imaging Linear Accelerator (MRI-Linac).

Prostate cancer is the second most frequently diagnosed cancer and the sixth leading cause of cancer death in males, accounting for 14 % (903, 500) of the total new cancer cases and 6 % (258, 400) of the total cancer deaths in males in 2008 (Jemal et al. 2011). Radical prostatectomy, external photon beam irradiation, and brachytherapy implantation are the usual methods for treating carcinoma of the prostate. The decision of which procedure, or which combination of procedures, to use depends on a number factors such as stage, grade, and the pre-treatment prostate-specific antigen (PSA) concentration. Radioactive brachytherapy seed implants are generally used for early stage cancers, either as a monotherapy (100 % of the prescribed dose) or in conjunction with external beam radiation therapy (67 % of the prescribed dose, referred to as a boost) (Khan and Gibbons 2014). The permanent transperineal interstitial placement of ^125^I seeds is a popular choice for low dose rate brachytherapy of prostate cancer. The deposited-seed positions are imaged and the plan optimized in real time throughout the procedure. The dose distribution is updated dynamically based on the actual positions as the seeds are deposited. The clinical and technological improvements that have emerged over the last decade in low dose rate prostate brachytherapy such as the use of ultrasound and computed tomography-based treatment planning systems has led to a resurgence in the use of the technique for localized prostate cancer (Phan et al. 2009).

The purpose of radiotherapy is to safely, accurately and efficiently deliver radiation to treat various types of cancers. Recently, a number of radiation incidents in various countries have been reported (Mayles 2007; Williams 2007; Derreumaux et al. 2008; Shafiq et al. 2009). The examples discussed in these reports demonstrate that newly developed radiation treatment techniques, and their implementation into the clinic, require an increasing level of alertness to verify the safe and accurate delivery of the prescribed treatment. To address these issues comprehensive quality assurance (QA) programs have been introduced. These programs should verify the correct functioning of all components in the radiotherapy chain including the treatment planning system (TPS)—against known tolerances—and treatment delivery system. In addition to these QA programs of the separate components required for a patient treatment, additional pre-treatment verification checks for individual patients are often performed using, for instance, independent dose or monitor unit (MU) calculation programs, and various QA devices such as ionization chamber and diode arrays.

A dosimeter is defined as a device that provides a reading that is a measure of the average absorbed dose deposited on its measuring body by ionising radiation. Codes of practice in the United Kingdom (Lillicrap et al. 1990; Thwaites et al. 2003) define how the dosimetric calibration of clinical beams is undertaken with ionization chamber recommendations. While there are many types of radiation dosimeters available, there is no single dosimeter that can detect all the different forms of radiation, and consequently, different types of dosimeters may be required to detect different forms of radiation depending on the application. Based on the application, an informed decision will be required for the type of dosimeter to be used.

In vivo dosimetry is the most direct method for monitoring the dose delivered to the patient receiving radiation therapy. When performed early in treatment, as a supplement to the clinical quality assurance (QA) program, simple in vivo measurements are an additional safeguard against major setup errors and calculation or transcription errors that were missed during the pre-treatment chart check. In the absence of errors, routine in vivo measurements uniquely document that treatment was delivered correctly within a user-specified tolerance. Unlike other QA methods, in vivo dosimetry checks the dose delivered to the patient rather than the individual components prior to treatment. There is a growing interest in the need to perform such in vivo measurements in part owing to increasing awareness of the potential risks associated with incorrect delivery or planning of radiation treatments, and because of the use of increasing complex delivery techniques such as intensity-modulated radiation therapy (IMRT) and VMAT, and the move towards more hypofractionated treatments delivered with large doses per fraction.

The initial motivation for performing in vivo dosimetry (IVD) in brachytherapy was mainly to assess doses to organs at risk (OAR) by direct measurements, because precise evaluation of OAR doses was difficult without 3D dose treatment planning. With 3D image-guided brachytherapy now the norm, it is now possible to calculate 3D tumour and OAR doses as well as dose volume histogram (DVH) parameters by the treatment planning system (TPS). However, there are situations where significant dose calculation uncertainties exists, in particular for low-energy photon-emitting sources (Iodine 125) and here IVD would contribute to more precise dose reporting. Furthermore, potential organ movement in between imaging and treatment contributes to uncertainties in delivered dose, and IVD have a potential role for identification of organ or applicator movements. Brachytherapy is typically administered with large doses per fraction (>5 Gy), which means that the consequence of a fractional error may be substantial when compared to typical external beam radiotherapy (EBRT) with delivery of smaller doses per fraction (e.g. 2 Gy). IVD is extremely important in brachytherapy as an independent method for detection of deviations or errors. However, for certain clinical situations TPS based dose calculations are currently associated with systematic deviations due to lack of corrections for heterogeneities and scatter conditions. This is particularly true for low-energy sources such as Iodine 125or 103Pd used in permanent implants of prostate. Some dosimetry systems define specific points or volumes for dose reporting for individual patients such as the American Brachytherapy Society (Davis et al. 2012) and this kind of reporting is important for patient related outcome measures (PROMS). In the situation where the tumour and OARs move and/or get deformed, as is the case during permanent prostate implants, IVD can provide additional information about the dose delivered to the tumour and/or OAR for the given patient. Future developments would have to be focused on the back end of the detection system utilised to process the data (i.e. the readings of the detector) with automatic data display, requiring less manpower, with no post data analysis or special expertise needed. Furthermore, to be successful for widespread use, any new technological innovation for such systems will have to be well integrated into the routine clinical application being targeted. Given existing alternatives for accurate brachytherapy dosimeters, future requirements will likely be made on in vivo dosimetry systems that can monitor the dose in real time, are sensitive to displacements of all applicators involved in the treatment, can identify organ motion, and that can provide immediate alerts of any potential gross error during ongoing treatments which alerts the clinician. The most common clinical in vivo dosimetry systems used are thermoluminescent detectors (TLDs), metal-oxide semiconductor field effect transistors (MOSFET), diodes, film and electronic portal imaging devices (EPIDs). These systems have advantages and disadvantages based on their application and are fully reviewed by Mijnheer et al. (2013). Not long after their clinical introduction for setup verification, it was realised that EPID images contain dose information. Consequently, several groups investigated the dosimetric characteristics of various types of EPIDs (Elmpt et al. 2008). Interest and development in EPID dosimetry has been accelerated by the advantages of fast image acquisition, high resolution, digital format, larger EPIDs with improved technology (Mijnheer et al. 2013). However, such technology requires an increased investment in dosimetry and associated hardware to support it. With the ongoing development, and soon to be commercially released, MRI-Linac (Stefanowicz et al. 2013), there has been an increased focus on other forms of in vivo dosimetry systems, such as optically stimulated luminescent dosimeters, radioluminescence dosimeters, plastic scintillation dosimeters and implantable MOSFETs (Reynolds et al. 2013).

## Properties of radiotherapy dosimeters

The accuracy of any dosimeter measurement is the ratio of expected value to that of the actual measured radiation. The precision of dosimetry measurements specifies the reproducibility of the measurements under similar conditions and can be estimated from the data obtained in repeated measurements. High precision is associated with a small standard deviation of the distribution of measurement results (Podgorsak 2005).

Ideally, the dosimeter reading should be linearly proportional to the dosimetric quantity. However, beyond a certain dose range a non-linearity generally sets in. The linearity range and the non-linearity behaviour depend on the type of dosimeter and its physical characteristics. In general, a non-linear behaviour should be corrected for. A dosimeter and its reader may both exhibit non-linear characteristics, but their combined effect could produce linearity over a wider range.

Integrating systems measure the integrated response of a dosimetry system. For such systems the measured dosimetric quantity should be independent of the rate of that quantity. Ideally, the response of a dosimetry system at two different dose rates should remain constant. In reality, the dose rate may influence the dosimeter readings and appropriate corrections are necessary, for example, recombination corrections for ionization chambers in pulsed beams in external beam radiotherapy.

The response of a dosimetry system is generally a function of radiation beam quality (energy). Since the dosimetry systems are calibrated at a specified radiation beam quality (or qualities) and used over a much wider energy range, the variation of the response of a dosimetry system with radiation quality (called energy dependence) requires correction. Ideally, the energy response should be flat (i.e. the system calibration should be independent of energy over a certain range of radiation qualities) enabling the dosimetry system to be used with various energies in both external beam radiotherapy and brachytherapy. In reality, the energy correction has to be included in the determination of the quantity for most measurement situations. In radiotherapy, the quantity of interest is the dose to water (or to tissue). As no dosimeter is water or tissue equivalent for all radiation beam qualities, the energy dependence is an important characteristic of a dosimetry system. Energy dependence has implications both for calibration of the dosimeter and for conversion of detector reading into measured dose during patient measurements. Most commonly used detectors exhibit higher energy dependence in brachytherapy than in megavoltage beams, because the photoelectric effect causes an over-response in the brachytherapy energy range.

The variation in response of a dosimeter with the angle of incidence of radiation is known as the directional, or angular, dependence of the dosimeter. Dosimeters usually exhibit directional dependence, due to their constructional details, physical size and the energy of the incident radiation. Therapy dosimeters are generally used in the same geometry as that in which they are calibrated. Directional independence is a very important characteristic in the field of LDR (low dose rate) brachytherapy due to the individual Iodine-125 seed placement which results in various orientations.

Since the dose is a point quantity, the dosimeter should allow the determination of the dose from a very small volume (i.e. one needs a ‘point dosimeter’ to characterize the dose at a point). The position of the point where the dose is determined (i.e. its spatial location) should be well defined in a reference coordinate system. Environmental variations such as temperature, pressure and humidity can cause variations in dosimeter readout; corrections to the measured dose may need to be implemented with a reference reading subtracted from measured reading.

Dosimeters should be rugged, easy to handle and not affected by the radiation itself. Dosimeters can be either active or passive types. Active dosimeters measure radiation exposure in real time with immediate results of radiation detected. Passive dosimeters do not provide real-time results, this is provided post-irradiation. Active type dosimeters provide numerous advantages over passive type dosimeters such as instant or direct reading of radiation levels which is now an ideal requirement. This paper presents a review of the state-of-the-art in optical fibre based sensor technology for radiation dosimetry in oncology. The work provides an introduction to the different techniques used and an overview of the developments in the application of these sensors to radiotherapy, specifically external beam radiotherapy and low dose rate (LDR) and high dose rate (HDR) brachytherapy. The authors have focussed their attention on prostate cancer and where possible an example of the sensor type implemented in a prostate cancer treatment environment is provided.

## Optical fibre dosimeters

Optical fibre dosimeters can have many advantages over existing systems such as TLDs, diodes or MOSFETs. A significant advantage is that optical fibres are composed of silica (glass) or plastic resulting in a material perfectly suited for the use in an MRI environment, as they are non-magnetic and do not cause interference of the image, immune to intense magnetic fields and radiofrequency (RF) present in this environment (Raaymakers et al. 2009). Optical fibres can also be summated together (multiplexed) to a single controller which in turn can form an array of detectors for 2d measurements. The development of radiation-resistant fibres has also meant that optical fibres can be utilised in areas of high levels of radiation. The small size of optical fibre sensors offers significant advantages for application in prostate brachytherapy. The small dimensions of the sensor (as low as 250 µm diameter) allows it to be easily guided within existing brachytherapy equipment; for example, within the seed implantation needle for direct tumour dose analysis, in the urinary catheter to monitor urethral dose, or within the biopsy needle holder of the transrectal ultrasound probe to monitor rectal wall dose. The measured radiation dose can be used to verify the calculated dose distribution that describes the treatment received by the patient. The availability of real-time radiation dose measurements during the brachytherapy procedure will allow for optimisation of the brachytherapy seed insertion during the procedure and result in high quality treatments. The quality of a brachytherapy treatment is directly linked to patient survival and outcomes (Hinnen et al. 2010). Radiation dosimetric methods are used for the estimation of dose absorbed by radiation in a detector material using either thermoluminescence (TL) technique or optically stimulated luminescence (OSL) technique or radioluminescence (RL) or any other technique using passive solid state detectors.

### Luminescence dosimetry

Luminescence occurs when a material is subjected to radiation and that material absorbs some of the radiation and as a result, emits light with a different wavelength. The wavelength of the emitted light is dependent on the luminescence material. Different types and forms of radiation can be used to excite a material and it is these types of radiation which give rise to different types or methods of luminescence. These are thermoluminescence (excitation due to heat), photoluminescence (excitation due to optical or ultra violet light) and radioluminescence (excitation is due to alpha, beta, gamma or x-rays).

#### Thermoluminescence dosimetry

**Fig. 1:**
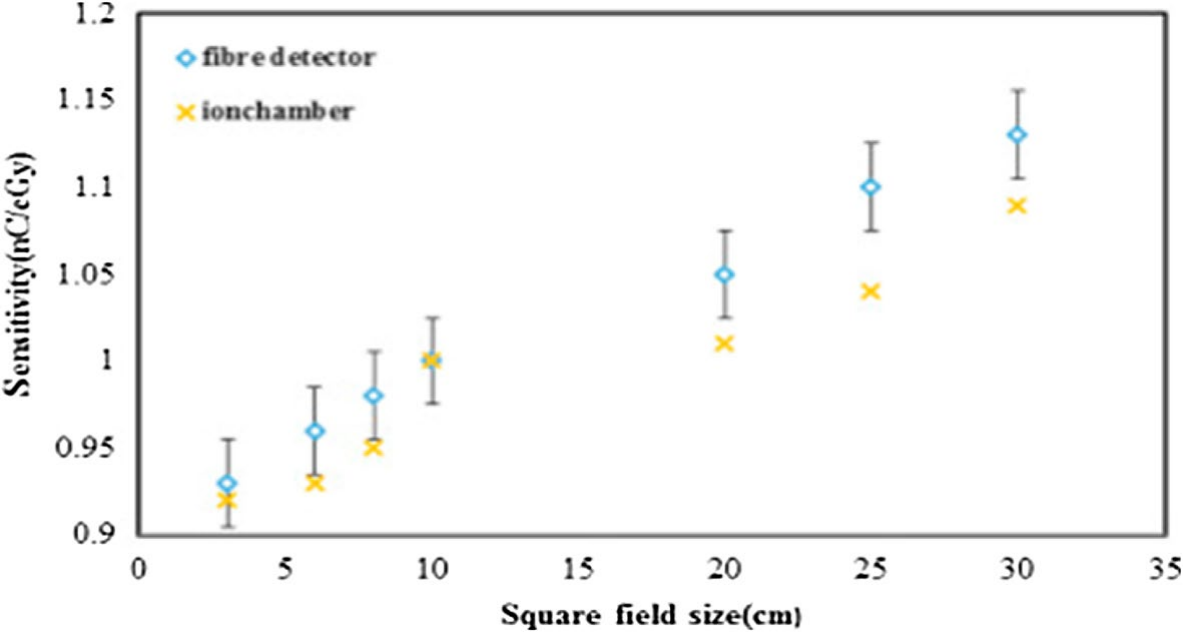
Sensitivity dependency of the 42 µm core size fibre on field size, measured for 6 MV X-rays. Reproduced from Entezam et al. (2016) with permission from Elsevier

Thermoluminescence (TL) is the emission of light from a solid due to heating, after it has previously been excited by radiation. When exposed to radiation, the TL material absorbs energy, which it then stores until it is heated. Germanium-doped silica (Ge-doped SiO_2_) type optical fibres have been demonstrated as potential novel forms of thermoluminescence dosimeters for radiation therapy dosimetry (Ramli et al. 2009). Abdul Rahman et al. (2012) performed an investigation in November 2011 into the ability of a high spatial resolution (~120 µm) Ge-doped SiO_2_ fibre thermoluminescence dosimeter to measure radiation. The optical fibres demonstrated good reproducibility (±1.5 %), at fixed dose rate, the dosimeters were found to produce a flat response of better than 4 % (1 S.D) of mean TL distribution and showed good linearity (*r* ^2^ = 0.998) of response up to a dose of 50 Gy for photon and electron beams. Entezam et al. (2016) reviewed the response behaviour, the fibres showed good reproducibility, energy, and field-size response, it was demonstrated that dependency on the field size for the most sensitive sample (the 42 µm core size fibre) was determined for photons produced at 6- and 10 MV and field sizes of 3, 6, 8, 10, 20, 25 and 30 cm. For each field size, measurement was carried out for five fibre segments of the 42 µm core size fibre, the results being normalized for all field sizes using the obtained value for 10 × 10 cm^2^ field size (Fig. 1).

Fading, the reduction of TL response as a function of time post-irradiation, depends primarily on parameters such as storage temperature and radiation type. The analysis of fading for flat and cylindrical fibres, fabricated by members of this group has been determined by Ghomeishi et al. (2015). For flat and cylindrical fibres irradiated simultaneously to a dose of 8 Gy using 6 MV photons, 15 days post-irradiation the TL response for the flat and cylindrical fibres had reduced by 17 and 27 %, respectively, compared to that obtained a day after irradiation.

One advantage of Ge-doped Si optical fibre dosimeters is that they are water resistant, and therefore, it becomes possible to locate the fibre dosimeter within a particular tissue of interest; this suggests the possible use of Ge-doped SiO_2_ optical fibres in a variety of interface dosimetry situations, as in the application of radiation synovectomy (Karavida and Notopoulos 2010). The potential radiotherapy dosimetric applications for doped silica fibres as TLDs have been reviewed by Bradley et al. (2012). The use of Ge-doped silica fibres has also been proposed by Issa et al. (2012) in brachytherapy, the optical fibres dosimeters have been employed in obtaining doses at distances very close to the source (2 mm). Dose measurements, obtained for separations from 2 up to 20 mm, were found to be in good agreement with simulations of photon-mediated dose obtained using the DOSRZnrc Monte Carlo code with agreement within 3 and 1 % for the ^133^Ba and ^60^Co sources, respectively. A significant disadvantage of thermoluminescent dosimetry is that the dose information is dependent on the post-irradiation of the material and so real-time dosimetry is not possible.

#### Optically stimulated luminescence

In a similar process to thermoluminescent techniques, photoluminescence, or optically stimulated luminescence (OSL), emits the energy stored owing to irradiation, upon exposure to light. When the insulator or semiconductor is subjected to radiation, electron hole pairs are generated, defects in the OSL material trap these electron hole pairs. This illumination of the material, frees the trapped electron hole pairs with the result of luminescence from the material transmitted through the fibre and measured with a photomultiplier tube.

Carbon-doped aluminium oxide (Al_2_O_3_:C) (Yukihara et al. 2014) showed how the response from OSL is linear and independent of energy and dose rate, exhibits little fading, temperature dependent and is sensitive to light. Europium-doped potassium bromide (KBr:Eu) were reviewed by McKeever (2011). It was discovered that the signal from KBr:Eu is unstable due to fading at room temperature, rapid OSL decay and its simpler process of OSL production enables the material to be used in real-time monitoring of radiation. Enhanced aluminium oxide doped with carbon and magnesium (Al_2_O_3_:C,Mg) (Rodriguez et al. 2011) were investigated for optically stimulated luminescence (OSL) in radiation dosimetry. Findings show that the intensities of TL, OSL and RL signals of the samples were similar to that of regular carbon-doped aluminium oxide (Al_2_O_3_:C).

Dunn et al. (2013) commissioned optically stimulated luminescence dosimeters (OSLDs) as a replacement for thermoluminescence dosimeters (TLDs) for application within radiotherapy, a product from Landauer Inc. known as “nanoDots” showed supra-linearity, reproducible fading (3 %) and little signal depletion per readout (0.03 %). Marckmann et al. (2006) developed a novel idea to overcome Cerenkov radiation by coupling OSL (Al_2_O_3_:C) to the end of a polymethyl methacrylate (PMMA) optical fibre to result in simultaneous RL/OSL signals providing real-time radiation monitoring using RL and post-radiation using OSL. The characterisation of a fibre based Al_2_O_3_:C OSL dosimeter by Anderson et al. (2009) for its response to 192Ir, demonstrates the suitability of such a device for HDR brachytherapy. The system demonstrated excellent linearity in the tested dose rage (0–4.3 Gy), with reproducibility of approximately 1.3 %. It was also estimated that measurements with a 5–50 mm source to probe distance would be associated with a 5 % uncertainty.

#### Radioluminescence

##### Plastic scintillating fibre dosimeters

Scintillating fibres work by converting incident radiation energy into visible light, as they are exposed to X-ray radiation, electrons in the fibre are excited to higher energy levels through either Compton or photoelectric effect. The fibre core is doped with scintillating fluorescent particles, which fluoresce when irradiated by ionising radiation, and cladded with PMMA. A major advantage of these dosimeters in radiotherapy is their water equivalence making them an ideal dosimeter in radiotherapy dosimetry. However, recent studies (Buranurak et al. 2013) identified the effect of temperature on a fibre coupled organic plastic scintillator in applications such as external beam radiotherapy and brachytherapy. The study showed that the light yield in the peak regions of the scintillators decreases linearly with increasing temperature. Temperature coefficients of −0.15 ± 0.01 and −0.55 ± 0.04 % K^−1^ for blue BCF-12 and green BCF-60 from Saint-Gobain Crystals were, respectively, observed in the study. Another study (Beddar 2012) showed significant differences from measurements inside patients to those measurements in anthropomorphic phantoms due to the similar effects of temperature.

**Fig. 2:**
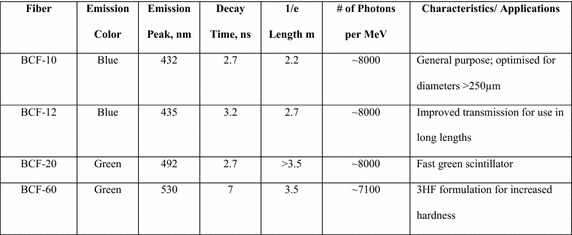
Four examples of scintillating fibres from Saint-Gobain Crystals (McKeever 2011)

Figure 2 shows the properties of four scintillating organic fibres which were used from Saint-Gobain Crystals (2015). These were BCF10, BCF12, BCF20 and BCF60. It shows that BCF10, BCF12 emit a blue colour whereas BCF20, BCF60 both emit a green colour with a resultant higher emission peak. They range from 0.25 to 5.00 mm in diameter, have a polystyrene core with fluorescent dopants and a PMMA cladding.

Suchowerska et al. (2011) showed a fibre optic scintillating dosimeter, consisting of a plastic scintillator coupled to an optical fibre for brachytherapy. These sensors were small enough (0.5 mm) to be inserted into a No. 16 French urinary catheter, to perform in vivo dosimetry to determine the urethral dose during high dose rate (HDR) treatment to the prostate. The background signal created by Cerenkov and fibre fluorescence was 0.1 % of the signal and the sensor had the capability of real-time readout. Klein et al. (Klein et al. 2012) demonstrated a plastic scintillating fibre (BCF-60) mounted onto an endorectal balloon to verify doses in vivo during intensity-modulated radiation therapy (IMRT) and volumetric arc therapy (VMAT) for prostate cancer. The sensor measured doses that correlated with ionization chamber measurements and it was found that treatment planning system calculations were within 1 % of expected values. A proposed dosimeter for in vivo dosimetry in HDR brachytherapy was investigated (Therriault-Proulx et al. 2013), whereby a single fibre with multipoint plastic scintillators was developed for Iridium-192 HDR brachytherapy treatment verification in a water phantom. It contained a three-point detector containing BCF-10, BCF-12 and BCF-60 scintillating elements. A comparison of measured doses at different source-to-detector distances were investigated, with the result that the system was suitable for measuring source position uncertainty to less than 0.32 ± 0.06 mm.

**Fig. 3:**
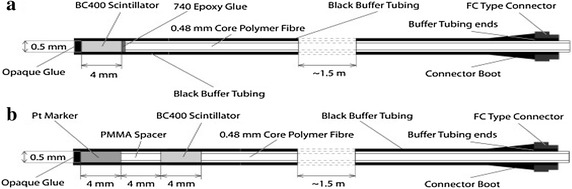
**a** Design of the BrachyFOD. **a** 4-mm-long 0.5-mm diameter Bicron BC400 scintillator is coupled to a 0.5-mm diameter (0.48-mm core) polymethyl methacrylate (PMMA) PMMA optical fibre. The entire dosimeter is covered with a black polyvinyl toluene PVT buffer tubing. **b** Design of the *R*FOD. The scintillator, spacer, and platinum radiopaque marker are all 0.5 mm in diameter and 4 mm long. Reproduced from Suchowerska et al. (2011) with the permission from Elsevier

A clinical trial of a plastic scintillating fibre dosimeter, BrachyFOD, (Suchowerska et al. 2011) enrolled 24 patients receiving HDR brachytherapy to the prostate. After 14 patients, the dosimeter design was improved for more accurate readings to improve clinical reliability: a dosimeter self-checking facility; a radiopaque marker to determine the position of the dosimeter, and a more robust optical extension fibre as depicted in Fig. 3. The results demonstrated a maximum measured dose difference of 9 % from the calculated dose from the TPS for the remaining patients in the trial indicating the importance of in vivo dosimetry in brachytherapy.

In a further study (Gagnon et al. 2012), the performance of a plastic scintillator BCF-60 was compared with a range of traditional small field dosimeters for stereotactic QA, the results compared output factors and dose profiles with a good level of agreement with diodes and EBT2 Gafchromic film. Currently, the only commercial optical fibre dosimeter for radiotherapy is the Standard Imaging Exradin W1 Scintillator (2014), a 1 mm core polystyrene-based fibre that is coupled to a PMMA optical fibre for transmission of the optical signal.

##### Inorganic scintillating fibre dosimeters

**Fig. 4:**
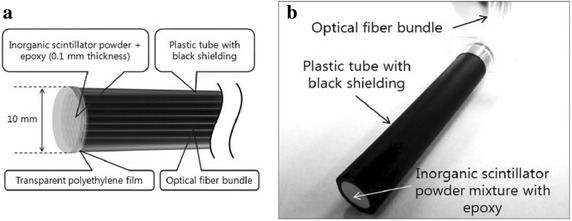
Fiber-optic sensor tip to detect beta rays. **a** Schematic diagram of sensor tip and **b** picture of sensor tip. Reproduced from Jang et al. (Jang et al. 2011) with permission from Elsevier

Inorganic scintillators are generally in crystal form grown at high temperatures. They are made of alkali halides, or oxides, and often require an activator impurity, e.g. Na(Tl), CsI(Tl). Due to the crystal form of the scintillator, it is possible to incorporate the material into the sensing region in a number of different ways, e.g. coating the fibre, coupling it to the time, or embedding it within the fibre. Distinct advantages include real-time dosimetry, small size and good spatial resolution. In evaluating the scintillation efficiencies of different phosphors, a radiation dosimeter (Jang et al. 2011) to detect tritium in real time was developed, composed of a scintillator material, an optical fibre bundle and alight measuring device, as illustrated in Fig. 4. Each scintillator interacts with electron or beta radiation and generates scintillation photons between 455 nm and 550 nm wavelength of light. Three kinds of inorganic scintillators were tested at different distances between the fibre optic sensor and source. These were Gd_2_O_2_S:Tb, cerium-doped YAG (Y_3_Al_5_O_12_:Ce) and CsI:Tl. The results show that the scintillation efficiencies of CsI:Tl, Y_3_Al_5_O_12_:Ce and Gd_2_O_2_S:Tb are 8, 5 and 15 %, respectively. The Gd_2_O_2_S:Tb type scintillator was found to give the greatest scintillation response of photons.

An optical fibre dosimeter has been developed by McCarthy et al. (2011; O’Keeffe et al. 2013) by coating the end of an exposed PMMA optical fibre, after the cladding has been removed, with Gd2O2S:Tb. The scintillating phosphor, supplied by Phosphor Technologies Ltd (2014) is mixed with an epoxy mix and injected into a cylindrical mould containing the exposed PMMA fibre optic core and allowed to cure. The radiation-sensitive scintillating material tip of the sensor fluoresces on immediate exposure to ionising radiation. The resultant emitted fluorescent light penetrates the PMMA optical fibre and propagates along the fibre to a distal scientific grade spectrometer from Ocean Optics (Dunedin, FL), where the intensity of the peak wavelength of the fluorescent light is measured. Initial characterization measurements of this sensor have been carried out, with its response being evaluated in water equivalent phantoms to assess whether it would be suitable for potential in vivo applications in either brachytherapy or external beam radiotherapy dosimetry. The results demonstrate that the fibre has a high sensitivity and good repeatability across a range of beam energies and types, and demonstrate a linear response from low doses of the order of centigray up to at least 16 Gy in a single delivery.

**Fig. 5:**
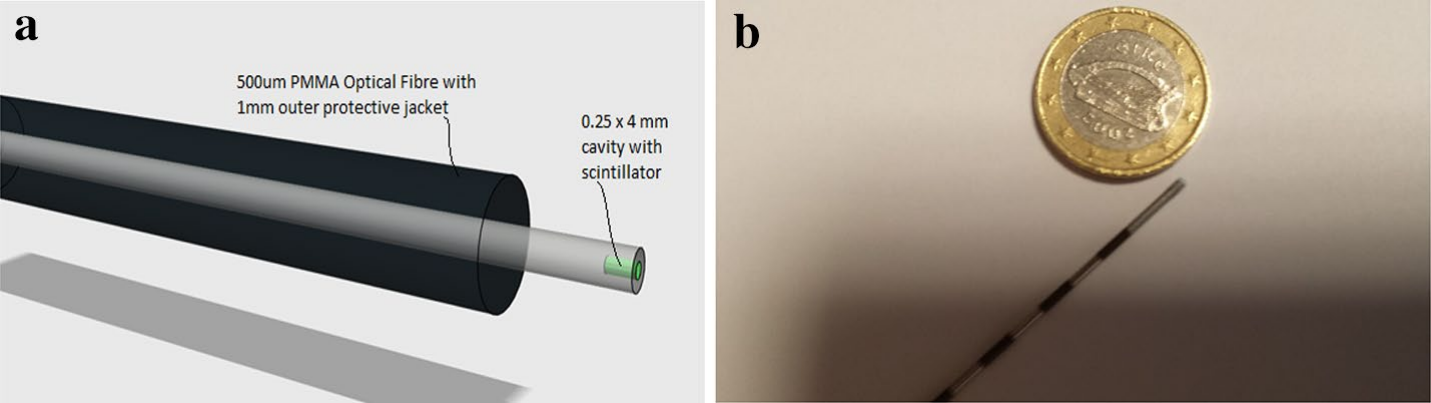
Optical fibre based radioluminescent radiation probe for in vivo brachytherapy.** a** Optical fibre based radiation probe for in-vivo brachytherapy.** b** Optical fibre sensor within brachytherapy needle for direct tumour dose monitoring. Reproduced from Woulfe et al. (2016) with permission from SPIE

An optical fibre sensor (Woulfe et al. 2016) based on radioluminescence, whereby radiation-sensitive scintillation material is embedded in the core of a plastic optical fibre, is illustrated in Fig. 5. Three sensors were fabricated, using different inorganic scintillators, identified as being most suitable for brachytherapy applications: thallium doped caesium iodide (CsI:Tl), terbium doped gadolinium oxysulphide (Gd_2_O_2_S:Tb, GOS) and europium-doped lanthanum oxysulphide (La_2_O_2_S:Eu, LOS). Terbium doped gadolinium oxysulphide (Gd_2_O_2_S:Tb, GOS) demonstrated the highest sensitivity to the ^125^I brachytherapy seeds (Woulfe et al. 2016). The sensor is designed for in vivo monitoring of the radiation dose during radioactive seed implantation for low dose rate brachytherapy, in prostate cancer treatment, providing oncologists with real-time information of the radiation dose to the target area and/or nearby critical structures. The radiation from the brachytherapy seeds causes emission of visible light from the scintillation material, which penetrates the fibre, propagating along the optical fibre for remote detection using a multi-pixel photon counter. The sensor demonstrates a high sensitivity to Iodine-125, the radioactive source most commonly used in brachytherapy for treating prostate cancer. The developed optical fibre based sensor has a number of significant advantages for application in brachytherapy. The small dimensions of the sensor allow them to be guided within existing brachytherapy equipment; for example, within the seed implantation needle (see Fig. 5b), in the urinary catheter to monitor urethral dose, or along the transperineal ultrasound probe to monitor rectal wall dose. This allows for real-time monitoring of the radiation dose to the target area or nearby critical structures. Furthermore, the construction of the sensors is such that they are completely biologically separate from their monitoring environment, and therefore, offer no possibility of contamination or other form of threat to their target operating environment, i.e. internal human tissue.

##### Fibre Bragg gratings

Fibre Bragg grating (FBG) based sensors work by monitoring the wavelength shift of the returned Bragg signal which changes as a function of the measured. The Bragg wavelength is related to the refractive index of the material and the grating pitch. The light incident on the grating reflects a narrow spectral component at the Bragg wavelength, and hence in the transmission spectrum this component is missing. Work (Mihailov 2012) has concentrated on developing radiation-resistant FBGs for use in temperature and strain measurement applications in nuclear environments.

The outcome of a project investigating the use of FBGs as high dose radiation sensors was first presented by Krebber et al. (2006). The work is based on the Kramer–Kronig dispersion relations, which can be used to show that an increase of attenuation has to be accompanied by a change of refractive index. The FBGs were written in a hydrogen doped Ge-doped fibre for wavelengths of 650, 820, 1285 and 1516 nm. The radiation induced refractive index change was calculated from the Bragg wavelength shift and a wavelength shift from 850 to 1216 nm was demonstrated to be independent of dose rate for radiation doses greater than 2 kGy. Although small changes in temperature are accounted for within the sensor system, the sensor requires a highly stable setup, with stress-free attachment of the FBG along with a constant, steady temperature. Fibre Bragg gratings, written in Ge‐doped silica fibres, have been shown to be capable of monitoring high‐radiation doses (Avino 2014).

## Cerenkov radiation (stem effect)

One of the primary disadvantages of using plastic scintillation detector systems is the generation of radiation induced light. This light is a form of noise in the received spectrum of light. It can be attributed to Cerenkov emission. A term known as the stem effect is used to describe Cerenkov light and other light which can be produced in a scintillation optical fibre dosimeter. Cerenkov radiation is generated when a charged particle passes through a medium with a velocity greater than that of light in that medium. Cerenkov emission only occurs when the refractive index of the material is greater than one. This emission of light generates a cone of light which spreads out at an angle. Cerenkov emissions are only produced above a certain energy threshold. For any radiation with energy below this threshold level, the presence of fluorescent light generated in the optical fibre should be considered. A number of different techniques have been shown to minimise the effects of Cerenkov light. These are background subtraction, filtering, and chromatic removal. These techniques help separate and remove the stem effect from the radiation signal (Archambault et al. 2006). The background subtraction is done by placing a second bare optical fibre next to the original sensing fibre. The bare fibre also produces the Cerenkov radiation and can be subtracted from the sensing fibre. The second method called spectral discrimination uses a colour CCD camera to measure both the scintillation and Cerenkov signal over different wavelength ranges. The Cerenkov is subtracted with the result that the scintillation signal is left behind. The third method is chromatic separation of the signal and Cerenkov emissions, through the use of optical filters. Another method of using a rigid air core light guide between the scintillator and the optical fibre was proposed to prevent Cerenkov light production (Law et al. 2006). Work by Law et al. have shown that both Cerenkov and fluorescent light generated in an optical fibre contribute a varying background signal that depends on the length of fibre exposed to radiation. They showed that the magnitude of the Cerenkov and fibre fluorescence is between 1 and 0.1 % of the signal, for the same source to fibre scintillator distance. Yoo et al. (2013) fabricated a novel fibre optic Cerenkov radiation sensor for measuring beta particles. Instead of using a scintillator, transparent liquids with various refractive indices were used as a Cerenkov radiator which served as a sensing material. The results showed that the amount of Cerenkov radiation due to low energy beta particles increased as the refractive index of the Cerenkov materials was increased.

## Dosimeter characteristics summary

An overview of the various types of dosimeters is depicted in Table 1 characterising their applications with emphasis on both advantages and disadvantages.

**Table 1:** Dosimeter characteristics summary

Dosimeter	Advantages	Disadvantages	Characteristics
Thermoluminescence dosimetry (TLD)	Used in Clinical QA dosimetry Linear from 0.01 Gy to 300 Gy Can be made small for point dose measurements Low cost and come in different sizes Monitors beta, gamma, X-ray and neutron radiations. Corrections for each type are needed	No real time measurement. Readout time can be consuming Supralinear from 300 Gy to 1 kGy TLD devices are re-useable but suffer from sensitivity with repeated use	Dose range: 0.10 mSv–10 Gy Energy response: Beta (MAX): 0.766 MeV–5 MeV, Photon: 5 keV–6 MeV, Neutron (TLD): Thermal–6 MeV, Neutron (CR39): 200 keV–6 MeV
Plastic Scintillating fibre optic dosimeter	Little energy dependence Rapid measurement time of around 2 s Linear from 0.01 Gy to 1 kGy Small (1 × 1 × 0.2 cm^3^) Stable for days Reusable, re-readable (0.03 % signal loss over 190 readings. Can be optically reset using UV	Supralinear response >2 Gy Sensitivity to light and temperature during irradiation and readout Room temperature fading of the OSL signal. Only two materials are commercially used in OSL dosimetry:Al2O3:C and BeO	Useful from 0.01 Sv to 100 Sv for X-ray and gamma radiation
Inorganic Scintillating fibre dosimeter	Used in radiotherapy type applications Can measure small volumes Long distance transmission, Immunity to electromagnetic interference	Effects of Cerenkov radiation Temperature dependent	Used for photon energies above 100 keV
Phosphor coated fibre	Used in radiotherapy and low dose personnel dosimetry applications Low cost and easy manufacture High scintillation efficiency 3.23 % variation detected at 90 kV 50 μA Accuracy of 2 % found under 15 MV 100 MU radiotherapy testing	High radiation exposure (50 Gy to 500 Gy) induces significant permanent attenuation in plastic optical fibres	Range from 50 kV to 15MV X-ray

## Conclusions

Radiation dosimetry has gained huge interest from many scientific disciplines and has led to the development of a huge number of publications across all the different types of dosimeter interests. This paper reviews a number of optical fibre based methods of dosimetry for radiation monitoring in prostate cancer therapy. Their small size, lightweight and flexibility have allowed their dosimetric performance to be demonstrated successfully in a range of measurement scenarios and have been demonstrated as being particularly effective for in vivo dosimetric verification of treatments such as HDR and prostate seed brachytherapy. Table 1 summarises the main characteristics of the principle radiotherapy dosimetry techniques based on optical fibres. Radioluminescence is the predominant technique used with RL showing the most potential within the area of in vivo dosimetry. PSDs have demonstrated significant advantages in the area of radiotherapy dosimetry, owing to their near-water equivalence, and this is reflected in the increasing number of publications in this area. Commercial interest has also focused on PSDs with the first optical fibre dosimeter for radiotherapy applications to reach the market, the Exradin W1, based on this technology, and further PSDs are also set to enter the market shortly. However, the recent reports on temperature dependence of this type of dosimeter must be investigated, and if necessary, accounted for, to ensure the reliability of the measurements.

Given existing alternatives for accurate brachytherapy dosimeters, future advancements will likely be made on in vivo dosimetry systems that can monitor the dose in real time, are sensitive to displacements of all applicators involved in the treatment, can identify organ motion, and that can provide immediate alerts of any potential gross error during ongoing treatments. Multiplexing of existing single-point optical fibre sensors will also play an important role in the future, allowing for volumetric dose measurements. Recently, it has been shown that it is possible to develop detectors composed of multiple different colour PSDs (mPSD) using a single optical transmission line and capable of measuring the dose accurately (François et al. 2012). Insertion of additional detector catheters may be performed with the purpose of optimizing detector positioning, e.g. by inserting an additional interstitial needle for housing a dosimeter probes in the tumour region. A smaller invasive impact may be achieved by inserting a sterilized dosimeter in the urethral catheter for urethral dose measurements. With a gamma analysis, an error criterion would be based not only on a dose difference but could also combine it with a distance-to-agreement criterion. In such an analysis, the upfront knowledge of the geometrical uncertainties of the detector and source positioning could be incorporated into the model to decide on relevant distance-to-agreement criteria.

It is the opinion of the authors that optical fibre sensors have an important role to play in in vivo dosimetry in radiotherapy for prostate cancer, providing direct real-time monitoring of the radiation delivered to the prostate and nearby critical structures, such as the urethra and rectal wall.
